# Inverse Transient Analysis for Classification of Wall Thickness Variations in Pipelines

**DOI:** 10.3390/s131217057

**Published:** 2013-12-11

**Authors:** Jeffrey Tuck, Pedro Lee

**Affiliations:** Department of Civil and Natural Resources Engineering, University of Canterbury, Private Bag 4800, Christchurch 8041, New Zealand; E-Mail: pedro.lee@canterbury.ac.nz

**Keywords:** transient, pipelines, water hammer, wall thickness, wavespeed, deterioration

## Abstract

Analysis of transient fluid pressure signals has been investigated as an alternative method of fault detection in pipeline systems and has shown promise in both laboratory and field trials. The advantage of the method is that it can potentially provide a fast and cost effective means of locating faults such as leaks, blockages and pipeline wall degradation within a pipeline while the system remains fully operational. The only requirement is that high speed pressure sensors are placed in contact with the fluid. Further development of the method requires detailed numerical models and enhanced understanding of transient flow within a pipeline where variations in pipeline condition and geometry occur. One such variation commonly encountered is the degradation or thinning of pipe walls, which can increase the susceptible of a pipeline to leak development. This paper aims to improve transient-based fault detection methods by investigating how changes in pipe wall thickness will affect the transient behaviour of a system; this is done through the analysis of laboratory experiments. The laboratory experiments are carried out on a stainless steel pipeline of constant outside diameter, into which a pipe section of variable wall thickness is inserted. In order to detect the location and severity of these changes in wall conditions within the laboratory system an inverse transient analysis procedure is employed which considers independent variations in wavespeed and diameter. Inverse transient analyses are carried out using a genetic algorithm optimisation routine to match the response from a one-dimensional method of characteristics transient model to the experimental time domain pressure responses. The accuracy of the detection technique is evaluated and benefits associated with various simplifying assumptions and simulation run times are investigated. It is found that for the case investigated, changes in the wavespeed and nominal diameter of the pipeline are both important to the accuracy of the inverse analysis procedure and can be used to differentiate the observed transient behaviour caused by changes in wall thickness from that caused by other known faults such as leaks. Further application of the method to real pipelines is discussed.

## 1. Introduction

Pipeline deterioration is a significant problem for engineers aiming to avoid costly failures or plan rehabilitation of pipeline assets. Typical forms of deterioration in pipeline systems include: internal or external corrosion of pipe walls, loss of lining and development of tubercles. These processes can lead to failure of the system through leak development, blockage formation or pipeline bursts which can lead to costly unexpected shutdowns, fluid contamination or increased running costs. Identification of pipeline deterioration has historically been carried out through external visual inspections, meaning that the identification of internal damage was more difficult. The development of closed circuit television (CCTV) cameras has enabled visual inspection of pipe interiors, however its range is limited and assessments can only be made based on damage that can be visually identified. Other inspection techniques such as eddy current analysis, ground penetrating radar, magnetic flux leakage and pipeline inspection gauges (PIGs) have been developed for pipeline inspection. While these methods enable the gathering of good quality data, they can be very expensive to implement and are intrusive, requiring physical entry to a pipeline system, excavation or system shutdowns [1].

To overcome the limitations of these existing methods the concept of analysing unsteady pressure responses within pipeline systems has been of interest to many research groups and is commonly referred to as transient analysis. An unsteady pressure response in a pipeline system is affected by any structural or geometric variations within that system and, as pressure waves can travel many kilometres within a pipeline, analysis of unsteady pressure responses within a system can potentially provide continuous information about the condition of that pipeline. Many methods for fault detection through transient analysis have been proposed, for which summaries can be found in Colombo *et al.* [2]. One such method takes transient pressure measurements from strategically placed pressure sensors in a pipeline system. Then, the transient pressure response can be used to determine the condition and physical state of a pipeline through inversely calibrating a numerical model to match the response, hence theoretically replicating the pipeline. This method is known as inverse transient analysis (ITA) and was first proposed by Pudar and Liggett [3]. For ITA to be successfully carried out a good understanding of the unsteady fluid behaviour in complex systems is required.

Transient analysis was first investigated by Stephens *et al.* [4] for the purposes of internal wall condition assessments of pipelines. The authors showed that changes in the condition of wall lining in a 750 mm mild steel cement lined (MSCL) pipeline would create reflections which can be used to characterise wall deterioration. Stephens *et al.* [5] followed on with this research and presented an ITA method of condition assessment which divided the pipeline into 15 m long sections, then inversely selected one of five predetermined levels of pipe damage for each section in an attempt to replicate the transient response of the system. The results showed reasonable correlation between the damage predicted by the ITA method and damage determined through the commercially available methods; ultrasonic pipe wall inspections and visual closed circuit television surveys. Hachem and Schleiss [6] carried out laboratory investigations that aimed to detect deterioration of pipe walls by considering simulated weak sections in a pipeline. The analysis methods used combined fast Fourier transforms and wavelet analysis techniques to locate the weak pipe sections. The weak sections were represented by using different pipe materials over short 0.5 m lengths. The method enabled the location of a single weak section of pipe to be determined along with a fair approximation of the wavespeed. Gong *et al.* [7] presented a Time Domain Reflectometry (TDR) method for the detection of a deteriorated section in a single pipeline. The method calculates the characteristic impedance of a deteriorated section by considering the magnitude of the initial reflection, from which the wavespeed and wall thickness of the section can be calculated by considering the equation for wavespeed in a fluid filled pipe (Equation (8)) which can be found in [8]. The method is shown to produce accurate results for laboratory experiments and is computationally cheap, however it makes a number of assumptions that may limit its application to field based analysis. These assumptions include: that corrosion of the pipe wall only occurs internally and does not affect Young's modulus of the material; that corrosion is uniform in both radial and longitudinal directions; that no corroded material remains attached to the pipe wall and that the time of the induced head perturbation is less than the time it takes for the wave front to travel two lengths of the deteriorated section. Accuracy of the method is also subject to the operator's selection of reference data points.

To improve upon the versatility of these detection methods it is necessary to reduce the number of simplifying assumptions. This paper describes an ITA method which can account for variations in the wavespeed, diameter and length of a deteriorated section independently, thus reducing the number of assumptions to be made.

## 2. Modelling Theory

This investigation uses the Method of Characteristics (MOC) to solving the governing mass and linear momentum conservation equations for one dimensional unsteady pipe flow [8]:

(1) $$\frac{gA}{a^{2}}\frac{\partial H}{\partial t}+\frac{\partial Q}{\partial x}=0$$

(2) $$\frac{1}{gA}\frac{\partial Q}{\partial t}+\frac{\partial H}{\partial x}+h_{f}=0$$

where *H* is the head in the pipe, *Q* is the pipe discharge, *A* is the cross-sectional area of the pipe, *a* is the wavespeed, *g* is acceleration due to gravity, *x* is the distance along the pipeline, *t* is time and *h*_f_ is the sum of steady and unsteady frictional head losses. The derivation of these two equations assumes that both the fluid and the pipe behave in a linear elastic fashion. The equations can be solved using the MOC through confining the solution to a grid in the time and space domains by applying the following relationship:

(3) $$\frac{dx}{dt}=\pm a$$

where *dx* is the grid spacing in the along the length of the pipe and *dt* is the time step for the numerical solution.

Solving Equations (1) and (2) subject to the condition in Equation (3) gives two simultaneous equations which can be used to solve for the head (*H_P_*) and flow (*Q_P_*) at a grid point where the head (*H_A_*, *H_B_*) and flow (*Q_A_*, *Q_B_*) are known values at adjacent nodes in the previous time step:

(4) $$H_{P}=H_{A}-B\left(Q_{P}-Q_{A}\right)-RQ_{P}\left|Q_{A}\right|$$

(5) $$H_{P}=H_{B}+B\left(Q_{P}-Q_{A}\right)+RQ_{P}\left|Q_{B}\right|$$

where *B* is the characteristic impedance of the pipeline given by:

(6) $$B=\frac{a}{gA}$$

and *R* is the pipeline resistance coefficient, which can be calculated by:

(7) $$R=\frac{f\;dx}{2gDA^{2}}$$

where *D* is the nominal diameter of the pipe section and *f* is the Darcy-Weisbach friction factor. The additional effects of unsteady friction can be accounted for using the efficient approximation of Vardy and Brown [9] for smooth turbulent pipe flow presented in Vitkovsky *et al.* [10].

The MOC model described is coded in Fortran using a constant time step discretisation such that numerical dissipation and dispersion errors that arise with the use of interpolation methods are avoided. The constant time step discretisation method requires the space step to be altered between sections of pipe to account for a change in the wavespeed as specified by Equation (3). Changes in pipeline properties in the longitudinal direction such as diameter and wavespeed can be accounted for by altering the variables in Equations (6) and (7).

## 3. Numerical Analysis

This section examines the effect that changes in pipeline wall condition have on transient behaviour within a pipeline. Changes in pipe wall conditions can alter three key parameters. The first is a change in the nominal diameter (*D*), which can increase due to internal corrosion of the pipe wall and delamination of internal protective linings, or decrease where corrosion leads to tuberculation. The second parameter is the wavespeed (*a*) which can be affected by the delamination of protective linings, internal and external corrosion and deterioration of the pipe wall material. The delamination and corrosion lead to a reduction in effective wall thickness, while pipe wall deterioration results in a decreased Young's modulus. The third parameter is a change in the friction factor (*f*) caused by a change in relative roughness. For the purposes of this research the relative effect of changes in friction factor are considered to be small, therefore they are not accounted for.

**Figure 1.**
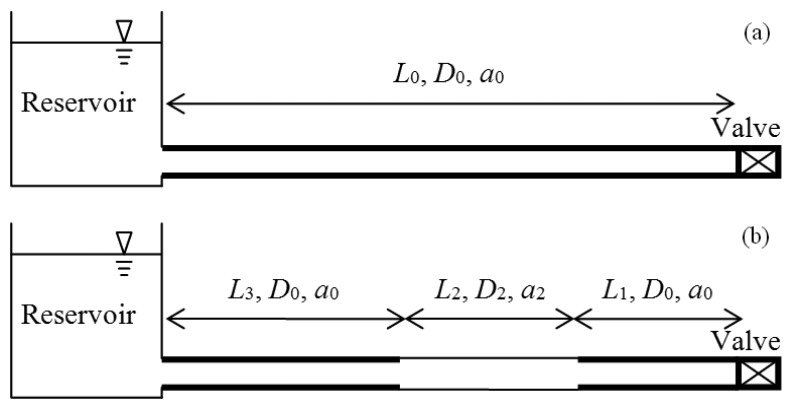
Schematic of reservoir, pipe and valve system for (**a**) an intact, fault free pipeline and (**b**) a pipeline with a section of reduced wall thickness.

Research presented in Tuck *et al.* [11,12] shows that a change in nominal diameter (*D*) or wavespeed (*a*) over a section of pipe can generate positive or negative reflections in the pressure response while also changing the fundamental period of oscillation for the system. To further illustrate these effects with a focus on the subject of pipe wall condition a numerical case study is used. This example considers a reservoir, pipe and valve system as depicted in Figure 1, where a 100 m long mild steel cement lined (MSCL) pipeline has lost the protective cement lining from a 20 m section located in the middle of the pipeline. The MSCL pipe considered has a nominal diameter (*D*) of 300 mm, steel wall thickness (*e_S_*) of 5 mm and cement thickness (*e_C_*) of 10 mm.

The wavespeed for a pipeline can be calculated by the wavespeed formula [8]:

(8) $$a=\frac{K/\rho }{1+[(K/E)(D/e)]c_{1}}$$

where the bulk modulus of the fluid (*K*) is taken as 2.14 GPa, the density of the fluid (*ρ*) is 999 kg/m^3^, *e* is the effective wall thickness, *E* is Young's modulus of the pipe wall material and *c*_1_ is a dimensionless parameter which accounts for constraint conditions on the pipeline and is taken as 1. To account for the relative strength of the cement lining an equivalent steel thickness can be calculated by the method in Stephens *et al.* [5] if it is assumed to be fully bonded to the steel:

(9) $$e=e_{S}+e_{C}(E_{C}/E_{S})$$

where Young's modulus of the pipe wall steel (*E_S_*) is 210 GPa and Young's modulus of the cement lining (*E_C_*) is 25 GPa.

**Figure 2.**
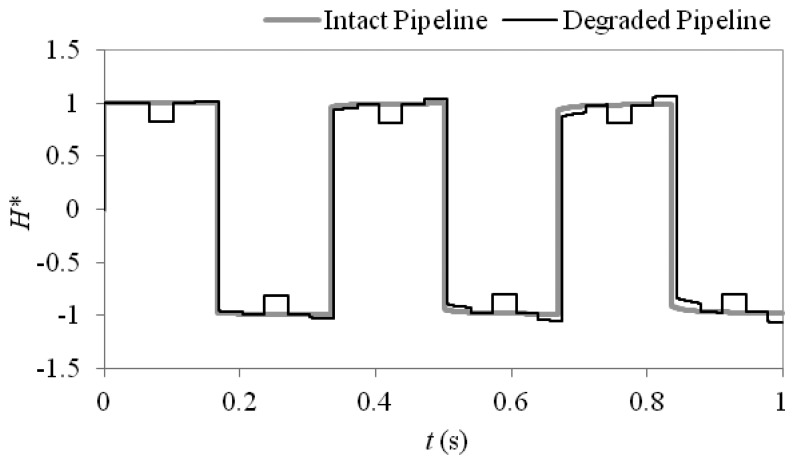
Comparison between an intact MSCL pipeline and a pipeline which has lost lining over a length of L_T_* = 0.2.

Using Equations (8) and (9) the wavespeed of the intact pipeline is calculated to be 1,197 m/s and the wavespeed over the section where the cement lining has delaminated is calculated to be 1,139 m/s. Figure 2 shows a comparison between the unsteady pressure responses from a pipeline with a section of delaminated cement lining and that of an intact pipeline as described above for this numerical case study. The unsteady pressure response is generated through instantaneous closure of the downstream online valve during fully turbulent flow conditions. The pressure head response *H*(*t*) has been shown at the downstream valve and is non-dimensionalised such that H* = *H*/*H_J_* where *H_J_* is the Joukowsky head rise. Following the initial Joukowsky head rise a reflection is observed from the downstream end of the degraded pipe section which gives a reduction in the pressure head. This reduction is caused by a decrease in characteristic impedance over the degraded section.

Following the initial drop in pressure head a reflection is observed from the upstream end of the degraded section which restores the pressure head back towards the observed values for the intact pipeline. Further secondary reflections are then observed before the pressure restoring reflection is observed from the upstream reservoir. The pressure response also shows a phase change, where the period of oscillation is increased for the system with the degraded pipeline.

## 4. Experimental Analysis

Laboratory experiments were carried out using the transient pipeline facility at the University of Canterbury to further investigate the effect that variations in pipe wall thickness have on unsteady fluid behaviour in pipelines. The experimental system consists of a 41.517 m long stainless steel pipeline with an external diameter of 76.2 mm and wall thickness of 1.5 mm. The pipeline is bounded by a pressure tank at the upstream end to represent a constant head reservoir and a discharge valve at the downstream end which can be rapidly closed to induce unsteady behaviour. The resulting pressure response is measured at the point of generation at a sampling rate of 10 kHz by high resolution Thermo Fisher Scientific, flush face, dynamic pressure transducers. The pressure transducers are accurate to within ±1% of the magnitude of the measured signal. This error is largely linear, thus will have little effect on the comparisons between numerical and experimental responses as the magnitude of the numerical response is based upon the Joukowsky head rise. Variations in pipe wall thickness were investigated through adding a section of pipe with a wall thickness of 3.65 mm and outside diameter equivalent to the existing pipeline. Thicker walled pipe was used for this experiment as sections with thinner walls than the existing pipeline were not commercially available and it was not feasible to replace the whole pipeline. The length of the thick walled section (*L*_2_) is 10.407 m and it is located at a distance (*L*_1_) of 16.550 m from the downstream valve. The wavespeed of the standard pipeline (*a*_1,3_) is experimentally determined as 1,180 m/s and the wave speed of the thick walled section (*a*_2_) is 1,315 m/s.

**Figure 3.**
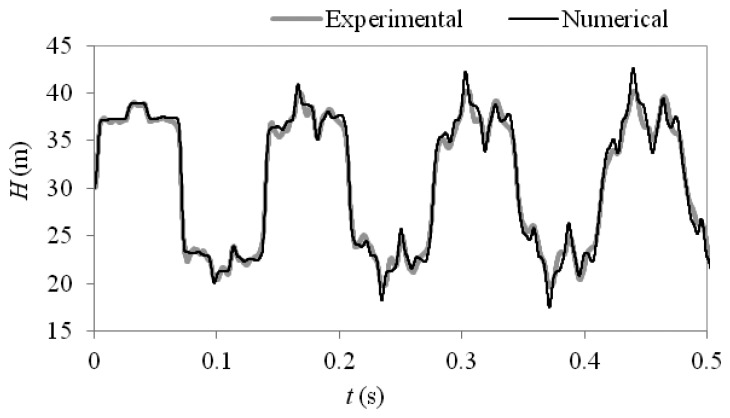
Comparison between numerical and experimental pressure responses for a pipeline with a thick walled section.

Figure 3 shows a comparison between the experimental pressure response and the MOC model over the first 3.5 periods of oscillation. The time step for the numerical model is taken as 0.0001 s to match the resolution of the experimental data which is sampled at a rate of 10 kHz. The comparison shows that the MOC model can provide a reasonable prediction of the transient behaviour and captures the complex reflection patterns induced by the thick walled pipe section. For this case the initial reflection observed from the thick walled section is positive as the nominal diameter is decreased and the wavespeed is increased which increases the impedance of the section, giving the opposite effect to that observed in the numerical case study. The differences observed between the numerical and experimental results can be attributed to differences in physical behaviour and in the experimental system which are not accounted for by the one dimensional model. These differences include fluid structure interaction, non-uniformities in the pipe materials due to manufacturing tolerances and additional damping from non-pipe elements such as the air bleed valves located on the experimental pipeline.

## 5. Inverse Transient Analysis

It has been shown in Figure 3 that a MOC model can produce an accurate representation of transient flow behaviour where variations in pipe wall thickness occur and the measured parameters are defined. This section considers the problem where the variables which define the “faulty” pipe section are unknown. To determine the unknown parameters the method of inverse transient analysis (ITA) can be used. This method aims to determine the values of the unknown parameters which achieve the best fit between predicted and measured pressure responses using the least squares analysis:

(10) $$s={\sum_{i=1}^{N}\left(H_{i}^{m}-H_{i}^{p}\right)}^{2}$$

where *s* is the residual error, N is the number of data points, $H_{i}^{m}$ is the measured pressure response and $H_{i}^{p}$ is the predicted pressure response. Equation (10) can be minimised using a genetic algorithm (GA) optimisation routine, where the GA used for this research is MATLAB's inbuilt “ga” function. In this paper the inverse analysis is carried out considering only data from a single measurement location, however it has been shown that further measurement locations can improve the results of the inverse analysis [13].

For this inverse problem the variables that are said to define the faulty section are its wavespeed (*a*_2_), diameter (*D*_2_), distance from the downstream valve to the fault (*L*_1_) and length of the faulty section (*L*_2_). The variables are assigned the following bounds; 800 m/s < *a*_2_ < 1,440 m/s, 0 m < *D*_2_ < 0.0762 m, 0 m < *L*_1_ < *L*_0_ and 0 m < *L*_2_ < *L*_0_ which are determined by allowing a lenient range of feasible values. To ensure that all solutions fall within the known pipeline geometry the following condition must also be met: *L*_1_ + *L*_2_ ≤ *L*_0_. Through selection of these variables and bounds the following assumptions are made; at most there is a single section of faulty pipeline, and the relative roughness does not increase significantly over the faulty section.

To improve the potential accuracy of the inverse analysis problem it is first necessary to determine an appropriate closure profile for the valve such that the head perturbation for the numerical response is similar to that of the experimental data. This step enables significantly more accurate results to be taken from the analysis and theoretically overcomes the problem discussed in Gong *et al.* [7], where discrepancies in the magnitude of a reflection can occur if the operation time of the transient generation device is greater than that required for a pressure wave to travel two lengths of a pipe section which causes an overlapping of the reflections. Where the profile of the generated head perturbation is appropriately matched the overlap of reflections from the front and rear of one or multiple sections can be accounted for in the simulations.

Table 1 shows the results for seven ITAs, the first five of which investigate the effect that simulation time has on the accuracy of the results. In the first case the simulation time is taken as just less than the time for the pressure restoring wave to return to the downstream valve, here the accuracy of the predicted values are poor, where the error is calculated as (|Measured Value–Predicted Value|/Measured Value) × 100%. The accuracy is poor as no reflection from the end of the system is observed for the wavespeed to be scaled against, so the fundamental period of the system is not matched. From the second case, which is simulated over a whole transient cycle, to the fifth case, which is simulated over four transient cycles, there is an observable trend towards increasing accuracy. This trend has also been noted for leak detection application by Vitkovsky *et al.* [14]. Case 5 shows that a solution to the inverse problem can be determined to a high level of accuracy with relative errors between 0.3% and 0.8% for each parameter. Two factors contribute towards the improved accuracy where the inverse analysis is simulated over a longer period of time. The first is that a larger number of transient cycles will lead to a greater importance being placed upon matching the fundamental period of oscillation for the system which is affected by extended variations in pipelines [11]. The second is that the extended variations create the same change in each period of the signal and is progressively reinforced with each cycle. It should also be noted that confining the numerical solution domain to a grid in space and time limits the theoretical accuracy of the ITA to *dx*/2, where *dx* is the space step used in the numerical model as determined by Equation (3). For the fixed time step discretisation method adopted in this analysis and a specified time step of 0.0001 s, the resolution of the MOC grid could account for an error of up to 0.36% for values of *L*_1_ and 0.63% for values of *L*_2_.

In an attempt to reduce the size of solution domain, cases 6 and 7 involved runs where changes in either the diameter or the wavespeed were excluded from the inverse calibration. An approximation such as this could prove useful where multiple faulty pipe sections are considered and can reduce the number of variables in the solution domain. This approximation is valid where the relative effect of one variable is much less than the others. The relative effects of diameter and wavespeed can first be considered by looking at the magnitude of the initial reflection from a fault. Scale analysis of Equation (6), which represents the pipeline impedance, indicates that for a general case the effect of a change in diameter is more important than that of a relative change in the wavespeed because the pipe area changes proportionally to the square of the diameter. For this specific set of experiments the percentage change in wavespeed is 11.44% while the change in area is similar at 11.66%. However the results presented in cases 6 and 7 indicate that for an ITA carried out over four periods (16*L*/*a*) the wavespeed variable becomes significantly more important in achieving accurate detection. This can be explained by considering the phase change exhibited in the system response. The phase shift in the system response is shown to be most affected by the relative change in wavespeed by Tuck *et al.* [11] for the given range. Cases 6 and 7 show that the solution domain can be simplified by excluding variations in the diameter or wavespeed from the ITA, though considering both variables separately significantly improves results as shown by comparison with case 5.

## 6. Conclusions

This paper has demonstrated how transient behaviour in a pipeline is altered by the presence of a degraded section of pipe which can be a precursor to pipeline failures. It has been shown that the degraded section can produce a reduction in wavespeed and changes in nominal diameter. These variations will alter the transient response of a pipeline, enabling transient analysis to be used to detect and classify degraded sections of a pipe. An inverse transient analysis method of fault detection has been implemented and shown to successfully determine the properties and location of a damaged pipe section. It has been demonstrated that the method can independently resolve changes in wavespeed and diameter over a wide solution space. This enables the method to be applied where prior information about a pipeline condition is minimal which is advantageous for field application of transient based condition assessment methods. The presented method has been evaluated using laboratory data which exhibit strong periodic behaviour. It is found that this periodic behaviour enables improvements in fault detection and classification accuracy. Where this periodic nature is not so strongly present, such as in large water distribution networks, improvements in accuracy could be achieved through a greater number of measurement points instead of increasing the duration of signal as considered here. Improvements in accuracy are also potentially achieved through increasing the sampling frequency and decreasing the modelled time step of the system response. For the experimental arrangement investigated it is demonstrated that the solution domain can be simplified through fixing the diameter variable in the ITA and varying only the wavespeed, length and location, however this reduces the accuracy of the fault detection method.

## Figures and Tables

**Table 1.** Inverse transient analysis results.

		***a*_2_(m/s)**	**% error**	***D*_2_(m)**	**% error**	***L*_1_(m)**	**% error**	***L*_2_(m)**	**% error**
**Measured Values**	Case	1315	-	0.0688	-	16.550	-	10.407	-
***t* < 2*L*/*a***	1	1054	19.8%	0.0623	9.4%	19.422	17.4%	7.945	23.7%
***t* = 4*L*/*a***	2	1336	1.6%	0.0706	2.6%	15.814	4.4%	12.211	17.3%
***t* = 8*L*/*a***	3	1360	3.4%	0.0717	4.2%	15.826	4.4%	12.568	20.8%
***t* = 12*L*/*a***	4	1339	1.8%	0.0704	2.3%	16.777	1.4%	10.457	0.5%
***t* = 16*L*/*a***	5	1326	0.8%	0.0683	0.7%	16.426	0.7%	10.442	0.3%
***t* = 16*L*/*a*, fixed *D*_2_=*D*_0_**	6	1320	0.4%	0.0732	-	17.543	6.0%	10.215	1.8%
***t* = 16*L*/*a*, fixed *a*_2_=*a*_0_**	7	1180	-	0.0590	14.2%	18.997	14.8%	12.325	18.4%
